# Breastfeeding: A Potential Excretion Route for Mothers and Implications for Infant Exposure to Perfluoroalkyl Acids

**DOI:** 10.1289/ehp.1306613

**Published:** 2013-11-26

**Authors:** Debapriya Mondal, Rosana Hernandez Weldon, Ben G. Armstrong, Lorna J. Gibson, Maria-Jose Lopez-Espinosa, Hyeong-Moo Shin, Tony Fletcher

**Affiliations:** 1 London School of Hygiene & Tropical Medicine, London, United Kingdom; 2 School of Environment and Life Sciences, University of Salford, Salford, United Kingdom; 3 Center for Environmental Research and Children’s Health, University of California, Berkeley, Berkeley, California, USA; 4 Spanish Consortium for Research on Epidemiology and Public Health (CIBERESP) and Center for Public Health Research (CSISP), Valencia, Spain; 5 School of Social Ecology, University of California, Irvine, Irvine, California, USA

## Abstract

Background: The presence of perfluoroalkyl acids (PFAAs) in breast milk has been documented, but their lactational transfer has been rarely studied. Determination of the elimination rates of these chemicals during breastfeeding is important and critical for assessing exposure in mothers and infants.

Objectives: We aimed to investigate the association between breastfeeding and maternal serum concentrations of perfluorooctanoic acid (PFOA), perfluorooctane sulfonate (PFOS), perfluorononanoic acid (PFNA), and perfluorohexane sulfonate (PFHxS). For a subset of the population, for whom we also have their infants’ measurements, we investigated associations of breastfeeding with infant serum PFAA concentrations.

Methods: The present analysis included 633 women from the C8 Science Panel Study who had a child < 3.5 years of age and who provided blood samples and reported detailed information on breastfeeding at the time of survey. PFAA serum concentrations were available for all mothers and 8% (*n* = 49) of the infants. Maternal and infant serum concentrations were regressed on duration of breastfeeding.

Results: Each month of breastfeeding was associated with lower maternal serum concentrations of PFOA (–3%; 95% CI: –5, –2%), PFOS (–3%; 95% CI: –3, –2%), PFNA (–2%; 95% CI: –2, –1%), and PFHxS (–1%; 95% CI: –2, 0%). The infant PFOA and PFOS serum concentrations were 6% (95% CI: 1, 10%) and 4% (95% CI: 1, 7%) higher per month of breastfeeding.

Conclusions: Breast milk is the optimal food for infants, but is also a PFAA excretion route for lactating mothers and exposure route for nursing infants.

Citation: Mondal D, Weldon RH, Armstrong BG, Gibson LJ, Lopez-Espinosa MJ, Shin HM, Fletcher T. 2014. Breastfeeding: a potential excretion route for mothers and implications for infant exposure to perfluoroalkyl acids. Environ Health Perspect 122:187–192; http://dx.doi.org/10.1289/ehp.1306613

## Introduction

Breast milk is the natural and optimal food for infants (World Health Organization 2012). The diverse and compelling advantages of breastfeeding for infants, mothers, families, and societies are well documented (Gartner et al. 2005). However, the presence of environmental chemicals in breast milk and their potential adverse effects on infant development and health are of concern. The transfer to breast milk and excretion during lactation, or the depuration (LaKind et al. 2001), of some persistent lipophilic organic pollutants such as polychlorinated biphenyls (PCBs) and polybrominated diphenyl ethers (PBDEs) has been extensively documented (Dekoning and Karmaus 2000; Hites 2004).In contrast to PCBs and PBDEs, which bind to lipids, perfluoroalkyl acids (PFAAs) strongly bind to the protein fraction in blood, notably to albumin (Voelkel et al. 2008). Thus, lactational transfer of PFAAs, including perfluorooctanoic acid (PFOA), perfluorooctane sulfonate (PFOS), perfluorononanoic acid (PFNA), and perfluorohexane sulfonate (PFHxS), is believed to be caused by binding to milk proteins (Butenhoff et al. 2006; Fromme et al. 2010; Kärrman et al. 2010). The protein concentration in human milk (9–11 g/L) is about 3–5 times lower than the protein fraction in the blood (35–50 g/L) and this may explain, in part, why PFAA concentrations are much lower in human milk than in maternal serum (Fromme et al. 2010; Kärrman et al. 2010; von Ehrenstein et al. 2009). The concentration in breast milk as a proportion of the concentration in maternal serum ranges from 3.4% to 11% for PFOA (Haug et al. 2011; Kim et al. 2011; Liu et al. 2011), 1% to 2% for PFOS (Fromme et al. 2010; Haug et al. 2011; Kärrman et al. 2007; Kim et al. 2011; Liu et al. 2011), 0.7% to 5% for PFNA (Kärrman et al. 2007; Kim et al. 2011; Liu et al. 2011), and 2% to 3% for PFHxS (Kärrman et al. 2007; Kim et al. 2011).

Although PFAA concentrations in maternal milk are relatively low, findings from several studies (Fromme et al. 2010; Kärrman et al. 2007; Tao et al. 2008; Thomsen et al. 2010) suggest that breast milk is the primary route of exposure for these contaminants for breastfed infants. A recent study estimated that breast milk contributed > 94% and > 83% of the total PFOS and PFOA exposure, respectively, in infants 6 months of age (Haug et al. 2011). Further, given this transfer of PFOA and PFOS to the infants, this route could be a significant excretion pathway for the lactating mothers. A longitudinal study measured concentrations in breast milk monthly in nine women and estimated that during 1 year of breastfeeding, the concentration of PFOA and PFOS in breast milk fell by about 94% and 37%, respectively (Thomsen et al. 2010). The correlation coefficient between breast milk and maternal serum concentrations was 0.99 for PFOA and 0.63 for PFOS. Strong correlations have also been reported by others (Fromme et al. 2010; Haug et al. 2011; Kärrman et al. 2007; Nakata et al. 2009).

We previously reported on the associations between maternal and infant serum levels of PFOA and PFOS in a population exposed to high concentrations of PFOA released by a chemical plant, in the Mid-Ohio Valley, USA (Frisbee et al. 2009; Mondal et al. 2012). For a small subset, 35 mother–infant pairs, our data allowed classification of mothers by the recorded “intention” to breastfeed as stated in birth records at the time of delivery. In infants 1–3 years of age, we observed higher PFOA and PFOS infant:mother serum ratios for mothers classified as having intended to exclusively breast feed (1.83 and 1.35, respectively) compared to ratios for mothers with breast and bottle or only bottle feeding intentions (1.14 and 1.12, respectively). This suggests that infants have higher serum levels, and/or that mothers have lower serum levels of these compounds as a consequence of breastfeeding. However, we did not have information on whether they carried out breastfeeding as per their stated intention at the time of birth, nor the duration of such feeding.

A subset of mothers enrolled in the C8 Health Project, whose infants were born shortly before or after the time of measuring PFAA serum concentrations in 2005–2006, were interviewed about their infant’s health and were asked to report information about initiation and duration of breastfeeding for the index infant. The aims of the present study are to investigate the association of breastfeeding with maternal serum PFOA, PFOS, PFNA, and PFHxS concentrations, and for a subset of the population, for whom we also have their infants’ measurements, to investigate the association of breastfeeding with infant PFAA serum concentrations.

## Methods

*Study population*. The details of the C8 Health Project study population, enrollment criteria, and consent procedures are described in a previous publication (Frisbee et al. 2009). All participants gave written informed consent before inclusion; parents or guardians provided consent on behalf of infants. The London School of Hygiene & Tropical Medicine (LSHTM) Ethics Committee approved this study. The C8 Health Project, conducted in 2005–2006, collected data from 35,788 females (of 69,030 participants), including pregnancy information and measurements of 10 PFAAs in serum from 23,815 women (67%). Methods used for measurement of serum PFAAs and quality controls are described elsewhere (Frisbee et al. 2009). Briefly, the technique used solid-phase extraction followed by reverse-phase high-performance liquid chromatographic separation and detection by tandem mass spectrometry. Estimates of precision for PFOA were within ± 10% for multiple replicates over the range of 0.5–40 ng/mL. Relative precision estimates for PFOS, PFNA, and PFHxS were similar to those for PFOA. The limit of detection (LOD) was 0.5 ng/mL for each PFAA, and observations below the LOD were assigned a value of 0.25 ng/mL.

The present study population comprises a subset of women who participated in the C8 Health Project; consented for further study; were either pregnant or, if not, had a singleton born child < 3.5 years of age at the time of blood collection in 2005–2006; were resident in West Virginia or Ohio; and had measured PFAA concentrations during the 2005–2006 survey. If they had had more than one child in that age range, only the youngest child was considered. Among the women who were eligible for the initial study (*n* = 1,543), 87% were successfully contacted by phone and invited for telephone interview in 2011, and among them 878 (65%) participated. Because the present study focused on the impact of lactation on PFAA concentrations, the subset included in the current analysis was restricted to the 633 women who were not pregnant at the time of blood collection. Among their 633 children < 3.5 years of age, 49 were infant participants in the C8 Health Project who also had serum PFAA concentrations measured in 2005– 2006. Therefore, the final study sample was 633 mothers and 49 infants.

*Breastfeeding*. In the questionnaire on breastfeeding, mothers were asked to recall whether the infant was breastfed or not. If the child was breastfed, the mother was asked how long the child was breastfed. From the information provided, we determined the duration of lactation and the time from the end of lactation to the date of blood sampling.

*Modeled PFOA uptake*. We categorized ongoing maternal exposure to PFOA as a consequence of production activities from the DuPont plant based on modeled PFOA annual total dose estimates derived using environmental, exposure, and pharmacokinetic modeling in conjunction with self-reported residential histories and tap water consumption rates, as described in detail elsewhere (Shin et al. 2011b). Briefly, information on plant operations and chemical releases was combined with information on environmental characteristics of the region through a series of linked environmental fate and transport models to estimate air and water PFOA concentrations during 1951–2008 (Shin et al. 2011a), and estimates of individual air and water intake rates were used to derive annual retrospective dose estimates for each participant (Shin et al. 2011b). For the present study, we used each mother’s modeled PFOA exposures to estimate her average exposure from the date of her index infant’s birth to the 2005–2006 survey. On the basis of the distribution among the 633 mothers (average, 316 μg/year), we categorized ongoing PFOA exposure as none (0 μg/year), low (1–50 μg/year), medium (51–1,000 μg/year), or high (> 1,000 μg/year).

Our sample size was too small to stratify infants’ data by exposure levels as was done for mothers’.

*Statistical analysis*. To estimate the impact of breastfeeding on maternal serum PFAA concentrations (*n* = 633), we performed linear regression analyses with measured serum PFAA concentrations [natural log (ln) transformed to normalize] as the outcome, and the duration of breastfeeding as the predictor. We also adjusted for other potential determinants of serum PFAAs, specifically parity prior to the index pregnancy (categorized as 0, 1, 2, or > 2) (Brantsater et al. 2013), maternal age at survey (categorized into 5-year intervals), and infant age at survey (categorized into 6-month intervals). In addition, for PFOA, the models were adjusted by water district of residence (Steenland et al. 2009). Duration of breastfeeding was modeled as a categorical variable (0, ≤ 6, 7–12, > 12 months) or as a continuous variable. For PFOA only, analyses were also stratified to investigate whether the association between lactation and serum concentrations is modified by ongoing maternal PFOA exposure.

We considered other potential confounders in the models: educational category for the mother (school, college or higher), reported household income in three categories and body mass index (BMI; categorized from underweight to obese). If the results didn’t change appreciably, being < 10% change in regression coefficients in the continuous models, they were not included in the final models for which results are presented.

To estimate the impact of breastfeeding on infant serum PFAA concentrations (*n* = 49), we used regression analyses of ln infant serum PFAAs as the outcome and the duration of breastfeeding as the predictor, adjusted for infant’s age and (for PFOA only) water district.

We used the statistical software package STATA for all statistical analyses (version 12; StataCorp, College Station, TX, USA).

## Results

In this study population, 60% (*n* = 379) of the mothers reported breastfeeding. The average age of the mothers (*n* = 633) and infants (*n* = 49) at the time of the 2005–2006 survey was 28 (range, 15–47) and 2.5 (range, 1.7–3.4) years, respectively. Table 1 shows the demographics of the study population. Women who breastfed were more likely to be older than women who did not and seemed to have higher income and more education, whereas there were no observed pronounced differences in BMI, parity, or water district between them. Table 2 shows the serum PFAA concentrations in the study population stratified by the breastfeeding categories. The geometric mean (GM) maternal and infant concentrations for this study population (all subjects) were similar to concentrations measured in the overall C8 Health Project study population among all mothers 15–47 years of age (*n* = 12,621) and among all infants 1–4 years (*n* = 236) (22.24, 14.78, 1.23, and 2.38 ng/mL respectively for PFOA, PFOS, PFNA, and PFHxS for mothers, and 39.75, 13.97, 1.30, and 3.70 ng/mL for PFOA, PFOS, PFNA, and PFHxS for infants, respectively). GM maternal serum PFAA concentrations were lower in mothers who breastfed the index child than in mothers who did not breastfeed the index child. Conversely, breastfed infants had higher GM serum PFAA (except for PFNA) than infants who were never breastfed. Unadjusted differences were modest except for serum PFOA concentrations in infants, which were more than twice as high in breastfed infants (GM = 49 ng/mL; 95% CI: 31, 79 ng/mL) than bottle-fed infants (GM = 22 ng/mL; 95% CI: 11, 42 ng/mL).

**Table 1:** Demographic variables for the mothers (*n* = 633) in this study (breastfeeding referring to the index child).

Demographic variable	Breastfed index child (*n *= 379)	Did not breastfeed (*n *= 254)	*p*‑Value
Maternal age (years) (mean ± SD)	30 ± 6	28 ± 6	0.000^*a*^
Average household income [*n* (%)]^*b*^			0.000
≤ $20,000	79 (23.2)	72 (32.4)
> 20,000 to ≤ 70,000	204 (59.8)	133 (59.9)
> $70,000	58 (17.0)	17 (7.7)
Missing	38	32
Years of school [*n *(%)]			0.000^*b*^
< 12 years	15 (4.0)	25 (9.9)
High school diploma or GED	93 (24.5)	103 (40.7)
College education	179 (47.2)	93 (36.8)
Bachelor’s degree or higher	92 (24.3)	32 (12.6)
Missing	0	1
BMI (kg/m^2^) [*n *(%)]^*c*^			0.211^*b*^
Underweight (16.5–18.4)	8 (2.1)	13 (5.2)
Normal (18.5–24.9)	16 (42.7)	95 (38.2)
Overweight (25–29.9)	98 (26.1)	63 (25.3)
Obese Class I (30–34.9)	65 (17.3)	40 (16.1)
Obese Class II (35–39.9)	30 (8.0)	23 (9.2)
Obese Class III (≥ 40)	14 (3.7)	15 (6.0)
Missing	4	5
Parity [*n* (%)] before most recent pregnancy			0.597^*b*^
Nulliparous	120 (31.7)	87 (34.3)
1	131 (34.6)	91 (35.8)
2	79 (20.8)	42 (16.5)
> 2	49 (12.9)	34 (13.4)
Water district [*n* (%)]			0.218^*b*^
Belpre, OH	25 (6.6)	18 (7.1)
Tuppers Plain, OH	70 (18.5)	38 (15.0)
Little Hocking, OH	60 (15.8)	36 (14.2)
Lubeck, WV	38 (10.0)	18 (7.1)
Mason, WV	45 (11.9)	45 (17.7)
Pomeroy, OH	8 (2.1)	10 (3.9)
Others^*d*^	133 (35.1)	89 (35.0)
^***a***^*t*-test. ^***b***^Chi-square test. ^***c***^BMI categories according to the World Health Organization (2000). ^***d***^Not on public water supply from six water districts at time of providing serum sample; most of whom have lived in one of the six districts previously.			

**Table 2:** Geometric means (95% CIs) of maternal and infant PFAA concentrations (ng/mL) stratified by reported breastfeeding category.

Breastfeeding category	Maternal serum PFAAs					Infant serum PFAAs				
	*n*	PFOA	PFOS	PFNA	PFHxS	*n*	PFOA	PFOS	PFNA	PFHxS
All	633	18.69 (17.13, 20.28)	12.33 (11.77, 12.92)	1.03 (0.99, 1.07)	1.86 (1.75, 1.97)	49	36.14 (24.87, 52.52)	13.21 (11.17, 15.61)	1.32 (1.18, 1.48)	3.79 (2.85, 5.04)
Breastfed	379	18.32 (16.36, 20.50)	11.63 (10.98, 12.31)	0.99 (0.94, 1.04)	1.83 (1.70, 1.97)	31	48.55 (31.17, 75.61)	13.54 (10.79, 17.00)	1.28 (1.11, 1.48)	3.95 (2.68, 5.83)
Child not breastfed	254	19.26 (16.80, 22.08)	13.48 (12.45, 14.58)	1.09 (1.03, 1.15)	1.90 (1.73, 2.08)	18	21.74 (11.21, 42.17)	12.65 (9.74, 16.43)	1.39 (1.14, 1.70)	3.53 (2.27, 5.48)

**Figure 1:**
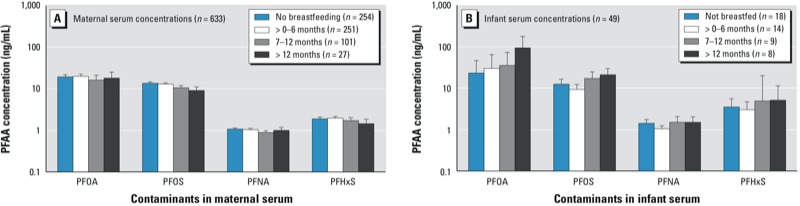
Unadjusted geometric mean (GM) maternal (*A*) and infant (*B*) PFAA serum concentrations (ng/mL) and 95% CIs stratified by duration of breastfeeding.

Among the 379 mothers who reported breastfeeding, the average duration was 3.5 months (range, < 1–35 months). At the time of 2005–2006 survey, 74 mothers were still breastfeeding and 305 had stopped breastfeeding, and for the latter the average time from end of lactation to survey was 11.2 months. Figure 1 shows maternal (*n* = 633), and infant (*n* = 49) serum PFAA concentrations stratified by the duration of breastfeeding. Although some trends of decrease in maternal serum concentrations and increase in infant serum concentrations with longer period of breastfeeding are suggested, as clearly observed for PFOS (maternal) and PFOA (infants), these are unadjusted means that may be confounded by other predictors such as age and water district.

Table 3 shows the age-adjusted association between maternal serum PFAA concentrations and the duration of breastfeeding, either categorical or as a continuous variable. Breastfeeding was associated with a reduction in maternal serum concentrations of 3% (95% CI: –5, –2%) for PFOA, and 3% (95% CI: –3, –2%) for PFOS, 2% (95% CI: –2, –1%) for PFNA, and 1% (95% CI: –2, 0%) for PFHxS per month of breastfeeding (though not significant for PFHxS). Except for PFHxS, maternal serum concentrations decreased monotonically as the duration of breastfeeding increased, although the confidence intervals overlap. When breastfeeding duration was modeled as a continuous variable, estimated decreases in maternal serum concentrations associated with 12 months of breastfeeding were 34% (95% CI: –44, –23%) for PFOA, 26% (95% CI: –34, –17%) for PFOS, 17% (95% CI: –24, –8%) for PFNA, and 12% (95% CI: –25, 1%) for PFHxS. However, only 7% (*n* = 27) of the breastfeeding mothers reported breastfeeding for more than 12 months. Sensitivity analysis controlling for household income, years of school, and BMI did not indicate confounding because these factors changed coefficients far less than 10% and so they were not included in the final models.

**Table 3:** Association between maternal PFAA serum concentrations and duration of breastfeeding.

Breastfeeding duration	*n*	PFOA		PFOS		PFNA		PFHxS	
		Percent change (95% CI)	*p*‑Value	Percent change (95% CI)	*p*‑Value	Percent change (95% CI)	*p*‑Value	Percent change (95% CI)	*p*‑Value
Not breastfed	254	Reference		Reference		Reference		Reference	
≤ 6 months	251	–5 (–18, 8)	0.448	–9 (–18, 1)	0.078	–8 (–16, 0)	0.044	2 (–10, 16)	0.703
7–12 months	101	–29 (–41, –13)	0.001	–24 (–34, –13)	0.000	–18 (–27, –8)	0.001	–13 (–27, 4)	0.125
> 12 months	27	–41 (–57, –17)	0.002	–39 (–52, –23)	0.000	–24 (–37, –7)	0.007	–15 (–37, 15)	0.298
Continuous (per month)	633	–3 (–5, –2)	0.000	–3 (–3, –2)	0.000	–2 (–2, –1)	0.000	–1 (–2, 0)	0.08
Estimates were based on models of maternal lnPFAA concentrations adjusted for parity (0, 1, 2, > 2), maternal age (categorical in 5-year intervals), and infant´s age (categorical in 6-month intervals). PFOA models were also adjusted for water district.									

Table 4 shows the percent change for maternal PFOA concentrations per month of breastfeeding stratified by degree of ongoing maternal exposure to PFOA. For mothers who were not exposed to PFOA above background levels after the index child’s birth, serum concentrations were 7% lower (95% CI: –11, –2%) with each month of breastfeeding. Among women with ongoing exposure, serum PFOA concentrations were also negatively associated with breastfeeding, but differences were less pronounced, and estimates were close to the null for women with medium or high levels of ongoing exposure. When the no- and low-exposure group and the medium- and high-exposure groups were each combined, serum PFOA concentrations were 4% lower (95% CI: –6, –3%) and 2% lower (95% CI: –4, 0%) with each month of breastfeeding, respectively.

**Table 4:** Association between maternal PFOA serum concentrations and duration of breastfeeding (per month), stratified by level of ongoing maternal PFOA uptake.^*a*^

None (background exposure only)			Low			Medium			High		
*n*	Percent change^*b*^ (95% CI)	*p*‑Value	*n*	Percent change^*b*^ (95% CI)	*p*‑Value	*n*	Percent change^*b*^ (95% CI)	*p*‑Value	*n*	Percent change^*b*^ (95% CI)	*p*‑Value
107	–7 (–11, –2)	0.006	299	–4 (–6, –3)	0.000	136	–2 (–5, 1)	0.258	91	–3 (–7, 1)	0.134
^***a***^Estimated as the average modeled exposure from the date of the index infant’s birth to the date of the 2005–2006 survey. ^***b***^Estimates were based on models of maternal lnPFAA concentrations with duration of breastfeeding (per month) as a continuous variable, adjusted for parity (0, 1, 2, > 2), maternal age (categorical in 5-year intervals), infant´s age (categorical in 6-month intervals), and water district.											

Age-adjusted results for infants are presented in Table 5. The observed estimates are unstable given the small number of observations. Nevertheless, for infants who were breastfed for ≥ 12 months (*n* = 8), serum concentrations were significantly higher compared with infants who were not breastfed for PFOA (141% higher; 95% CI: 4, 460%) and PFOS (71% higher; 95% CI: 9, 167%). Regression analysis of the infant PFAA concentrations on duration of breastfeeding modeled as a continuous variable predicted 6% (95% CI: 1, 10%) and 4% (95% CI: 1, 7%) increase in PFOA and PFOS infant serum concentrations, respectively, with each month of breastfeeding. Weaker relationships were suggested for PFHxS and PFNA, with statistically nonsignificant associations. From the continuous regression, estimated increases in infant serum concentrations associated with 12 months of breastfeeding were 96% (95% CI: 16, 231%) for PFOA and 64% (95% CI: 21, 122%) for PFOS.

**Table 5:** Associations between infant serum PFAA concentrations and duration of breastfeeding.

Breastfeeding duration	*n*	PFOA		PFOS		PFNA		PFHxS	
		Percent change (95% CI)	*p*‑Value	Percent change (95% CI)	*p*‑Value	Percent change (95% CI)	*p*‑Value	Percent change (95% CI)	*p*‑Value
Not breastfed	18	Ref		Ref		Ref		Ref	
≤ 6 months	14	13 (–46, 139)	0.728	–31 (–53, 1)	0.058	–24 (–43, 0)	0.052	–20 (–62, 70)	0.562
7–12 months	9	82 (–23, 334)	0.168	40 (–9, 115)	0.119	2 (–26, 41)	0.875	45 (–38, 240)	0.373
> 12 months	8	141 (4, 460)	0.041	71 (9, 167)	0.021	17 (–17, 63)	0.356	46 (–40, 255)	0.392
Continuous (per month)	49	6 (1, 10)	0.014	4 (1, 7)	0.002	1 (–1, 3)	0.157	2 (–3, 7)	0.289
Estimates were based on models of infant lnPFAA concentrations adjusted for infant´s age (categorical in 6-month intervals). PFOA models were also adjusted for water district.									

## Discussion

Breastfeeding is the preferred nutrition for the infant, but the presence of environmental chemicals in breast milk has gained increased attention in recent years. Understanding the elimination rates of chemicals from the mother during breastfeeding is critical for assessing exposure in mothers and infants (LaKind et al. 2001). Though the concentrations of PFAAs in breast milk have been widely measured in different populations (Fromme et al. 2010; Kärrman et al. 2007; So et al. 2006; Tao et al. 2008; Voelkel et al. 2008; von Ehrenstein et al. 2009), to the best of our knowledge very few data are available on the depuration rate of PFAAs due to lactation. We estimated that each month of breastfeeding was associated with maternal serum concentrations that were 3% lower for both PFOA and PFOS. Thomsen et al. (2010) estimated depuration rates of PFOA and PFOS due to breastfeeding by measuring PFOS and PFOA in milk samples collected from nine healthy primiparous mothers every month after birth up to 12 months, and estimated annual reductions of 94% for PFOA and 37% for PFOS. The large apparent difference in the excretion rate for PFOA between the Thomsen et al. study and the present study (94% vs. 34%) may be explained by relatively high PFOA exposures in our study population compared with the Norwegian mothers (Thomsen et al. 2010). Our data for mothers living in nonexposed areas are more comparable to the Thomsen et al. study; we estimated a 7% decrease in serum PFOA per month of breastfeeding, equivalent to a 60% decrease in PFOA concentrations after 12 months of breastfeeding. Recently, a 2–3% reduction in serum PFAA concentrations per month of breastfeeding (with PFOA having the highest estimated decline of 2.4%) was reported in a study of 487 Norwegian mothers enrolled in the MoBa (Mother and Child Cohort Study) cohort (Brantsater et al. 2013) based on measured concentrations during gestation and the total number of months of breastfeeding reported for all previous children. The estimated loss from lactation is in addition to loss from normal metabolism and net excretion, which has been estimated to be approximately 22–13% per year in adults following cessation of exposure, given a half-life of 2.3–3.8 years (Bartell et al. 2010; Olsen et al. 2007). Fei et al. (2010) reported that the duration of breastfeeding was negatively associated with serum PFOA and PFOS concentrations measured during pregnancy among 1,400 women enrolled in the Danish national birth cohort, but only among multiparous women. The authors suggested that their findings could be explained if women who breastfed previous children for a relatively long time were also more likely to breastfeed the child included in the cohort study for a long period, and if their previous lactation also resulted in lower concentrations of PFOA and PFOS during the index pregnancy. If so, their findings also would be consistent with those of the present study.

We can compare the observed depuration rate to that expected from average predicted excretion rates. Assuming average values of infant breast milk consumption of 700 mL/day (Thomsen et al. 2010) and partitioning of 6.2% between maternal serum and breast milk [i.e., midway between the lowest (3.4%) and highest (9%) estimates previously reported for PFOA] (Haug et al. 2011; Kim et al. 2011; Liu et al. 2011), we estimate that the amount of PFOA excreted in breast milk by the mother (nanograms per day) is 43.4 times the maternal serum concentration (nanograms per milliliter) (43.4 is 6.2% of 700 mL). The volume of distribution per kilogram body weight for PFOA in females has been estimated as 198 mL/kg (Butenhoff et al. 2004), and the average body weight of the mothers in the study population is 72.56 kg. The amount of PFOA in the mother’s body is thus estimated to be the product of volume of distribution, 14,366 mL, times the maternal serum concentration. Hence the proportional change in PFOA per day from mother’s body due to excretion via lactation is estimated to be 43.4/14,366, cumulating to an annual decrease of 33.1%, similar to what we have observed.

Another useful outcome of these results is to allow improvement in the exposure prediction model for the *in utero* exposure of these mothers’ infants. For a mother who provided blood samples after weaning, we can take this lactation-related reduction of PFOA into account for back estimating their serum levels during the pregnancy.

To best of our knowledge, this is the first study investigating the association between breastfeeding and infant PFAA serum concentrations. We estimated significantly higher serum PFOA and PFOS concentrations in infants breastfed for > 12 months compared with non-breastfed infants, though estimates were imprecise because they were based on only eight infants in the long-duration group. These results are subject to small sample size, and we observed that the associations between duration of breastfeeding and infant serum PFOS, PFNA, and PFHxS concentrations were not monotonic, with the lowest serum concentrations estimated for infants breastfed for ≤ 6 months, and for PFNA the estimates for the highest duration group were not clearly different from the null. Fitted as a continuous measure, each month of breastfeeding was associated with increase in 6% (95% CI: 1, 10%) and 4% (95% CI: 1, 7%) infant serum PFOA and PFOS concentrations, respectively—equivalent to an estimated increase over 12 months of breastfeeding of 96% (95% CI: 16, 231%) for PFOA and 64% (95% CI: 21, 122%) for PFOS. The increase in PFNA and PFHxS infant serum concentrations with each month of breastfeeding were not significant.

We have contrasted breastfeeding with formula feeding, and some of the water supplies were still PFOA contaminated to an extent in the years just before 2005. Thus, the association with breastfeeding may be under-estimated as a consequence of any exposure via tap water potentially used in mixing formula. Exposure to PFOA and PFOS in this population has fallen, and we have found a steep downward trend in serum levels since 2005. Thus, the degree of mother-to-infant PFOA and PFOS transfer has been consequently falling in this community.

This study benefited from the measured maternal serum concentrations over a range of durations of breastfeeding, but is not longitudinal, because repeated measured concentrations were not collected during the course of breastfeeding to allow a direct estimate of actual change. It cannot be ruled out that other processes occur during lactation which may affect excretion or blood volume and thus PFAA levels, or that women who choose to breastfeed also avoid food and drink sources with higher levels of contaminants. Nevertheless, our findings are consistent with those of two previous studies (Brantsater et al. 2013; Thomsen et al. 2010), which reported that breastfeeding was associated with lower serum PFAA concentrations in mothers. We found positive associations of breastfeeding > 12 months (vs. no breastfeeding) and serum PFOA and PFOS in infants; however, estimates were based on small numbers of observations and must be interpreted with caution.

## Conclusion

Our findings add to evidence suggesting that breastfeeding is an important PFAA excretion route for lactating mothers and exposure route for nursing infants. Each month of breastfeeding was associated with lower maternal serum PFAA concentrations. To our knowledge, we are the first to have estimated associations between breastfeeding and serum PFAA concentrations in infants, and although our findings were based on small numbers of observations, they provide preliminary evidence that breastfeeding for > 6 months may increase serum PFOS and PFOA concentrations in infants. Although breastfeeding can be an important PFAA excretion route for lactating mothers and exposure route for nursing infants, for most people, levels are very low and it is important to note that breast milk remains the optimal food for infants.
